# Is Laparoscopic Hepatectomy Safe for Giant Liver Tumors? Proposal from a Single Institution for Totally Laparoscopic Hemihepatectomy Using an Anterior Approach for Giant Liver Tumors Larger Than 10 cm in Diameter

**DOI:** 10.3390/curroncol29110652

**Published:** 2022-10-31

**Authors:** Hiroyuki Nitta, Akira Sasaki, Hirokatsu Katagiri, Shoji Kanno, Akira Umemura

**Affiliations:** Department of Surgery, Iwate Medical University School of Medicine, Morioka 028-3695, Japan

**Keywords:** giant liver tumor, laparoscopic hepatectomy, anterior approach

## Abstract

Background: The efficacy and safety of laparoscopic liver resections for liver tumors that are larger than 10 cm remain unclear. We developed a safe laparoscopic right hemihepatectomy for giant liver tumors using an anterior approach. Methods: Eighty patients who underwent laparoscopic hemihepatectomy between January 2011 and December 2021 were divided into a nongiant tumor group (*n* = 65) and a giant tumor group (*n* = 15) for comparison. Results: The median operating time, amount of blood loss, and length of postoperative hospital stay did not differ significantly between the nongiant and giant tumor groups. The sizes of the tumors and weights of the resected liver were significantly larger in the giant tumor group. A comparison between a nongiant group (*n* = 23) and a giant group (*n* = 12) treated with laparoscopic right hemihepatectomy showed similar results. Conclusions: Laparoscopic hemihepatectomy, especially that performed on the right side, for giant tumors larger than 10 cm can be performed safely. Surgical techniques for giant liver tumors have been standardized, and their application is expected to spread widely in the future.

## 1. Introduction

Laparoscopic hepatectomy was first reported in the early 1990s and has gradually spread as a minimally invasive surgery, with the first international consensus meeting held in Louisville in 2008 [1,2]. However, at that time, there was skepticism about the safety of major hepatectomy, and the preferred indication for laparoscopic hepatectomy was a peripheral site that was less than 5 cm and easily accessible [2]. Since then, appropriate respiratory and circulatory management and development of surgical techniques have enabled laparoscopic major hepatectomy to be performed safely at many hospitals, and recent international consensus meetings have confirmed the safety associated with the expansion of surgical indications [3,4,5,6].

Because the degree of difficulty of laparoscopic liver resection differs depending on the type of hepatectomy and tumor conditions, a difficulty scoring system was proposed by Ban et al. [7]. In this difficulty scoring system, the difficulty increases when the tumor diameter is 3 cm or more. There have been several reports on the results of laparoscopic liver resection for giant liver tumors of 5 cm or larger [8,9]. According to these reports, the operation time is extended and the blood loss is increased, but it can be completed safely. A report concerning a hepatocellular carcinoma (HCC) of more than 10 cm concluded that although the operation time was extended, there was no difference in blood loss and morbidity, and that it could be performed safely [10]. An open hepatectomy for a giant liver tumor requires a very big incision and, in some cases, thoracotomy. If laparoscopic hepatectomy can be safely completed for giant liver tumors, there is no question that the burden for the patient will be reduced because there will be less destruction of the body wall.

We performed laparoscopic hemihepatectomy for giant tumors larger than 10 cm using an anterior approach. For right hemihepatectomy in particular, a safe operation with controlled bleeding was made possible by creating a space on the right side of the inferior vena cava and compressing the liver parenchyma from the dorsal side. This surgical technique resulted in comparable perioperative surgical outcomes for giant and nongiant tumors.

## 2. Materials and Methods

### 2.1. Study Design

Between January 2011 and December 2021, 1041 patients underwent liver resections at Iwate Medical University Hospital. From the patient database of the hospital, a total of 154 patients undergoing hemihepatectomy for HCC, metastatic liver cancer, and benign disease, were identified, of whom 80 underwent totally laparoscopic hemihepatectomy (TLhH). This group included 15 patients with a tumor diameter of 10 cm or more. A comparison of the patients with tumor diameters of less than 10 cm (*n* = 65) and more than 10 cm (*n* = 15) who underwent TLhH was carried out. The patients did not undergo bile duct resection or lymphadenectomy. Patients with lesions involving the diaphragm or inferior vena cava were excluded.

### 2.2. Statistical Analysis

Variables analyzed included operation time, blood loss, resected tumor size, postoperative hospital stay, morbidity, and mortality. Morbidity and mortality were defined as those occurring within 90 days of surgery. Continuous data for each variable were represented by the mean. To compare the groups, Student’s *t*-test was applied to the continuous data and the χ^2^ test to the categorical data. *p* < 0.05 was considered to be statistically significant.

### 2.3. Surgical Procedure

#### 2.3.1. Right Hemihepatectomy

**Figure 1:**
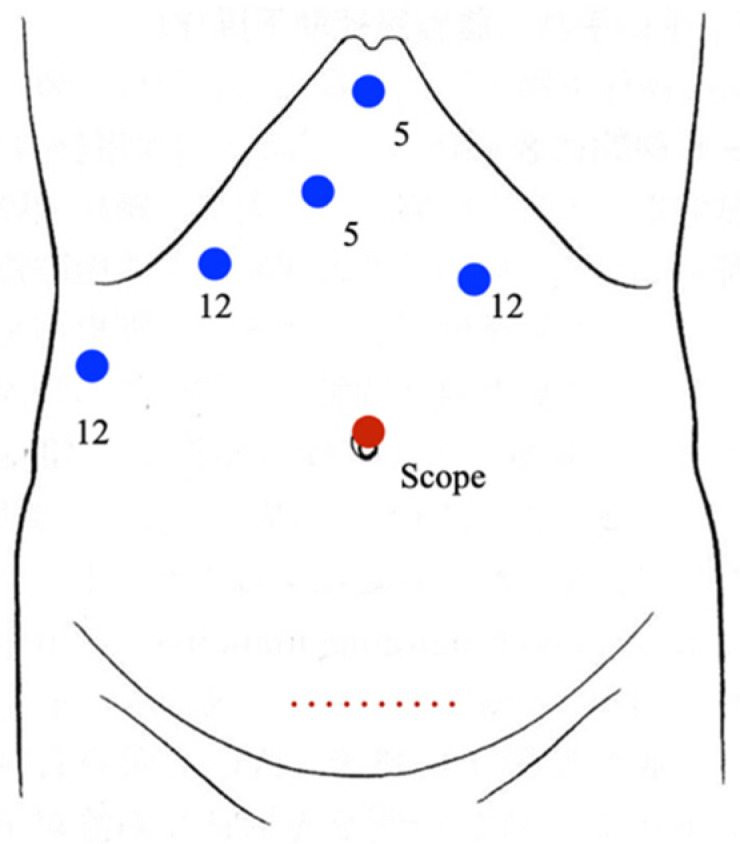
Trocar placement. The blue circle is the surgical port, and the red circle is the camera port. 5 and 12 are port diameters (mm).

The patient is in the supine position. A trocar for a laparoscope is inserted from the umbilical area to induce CO_2_ pneumoperitoneum (10 mmHg). Trocar placement is shown in Figure 1.

**Figure 2:**
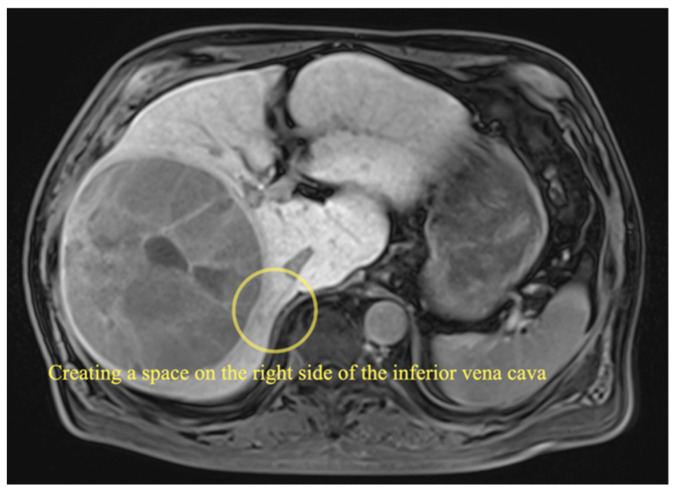
MRI image of giant HCC.

**Figure 3:**
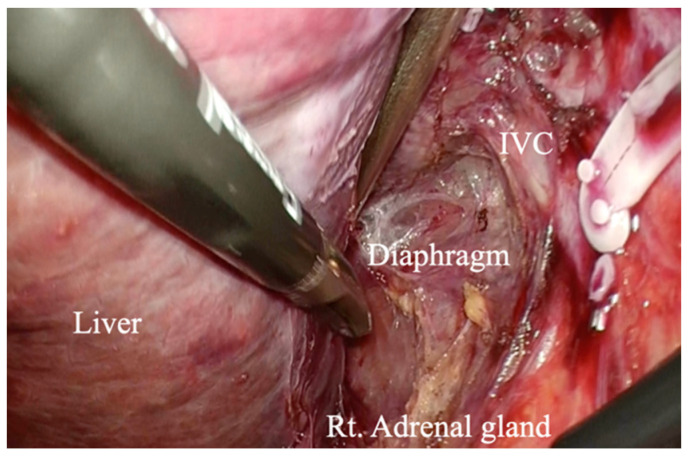
Intraoperative photo with a space created on the right side of the inferior vena cava.

Intraoperative ultrasonography is performed to ascertain the location of the tumor. Liver parenchymal transection is performed using an anterior approach [11]. A space is created on the right side of the inferior vena cava for dorsal retraction during parenchymal transection. (Figure 2 and Figure 3). The right inferior hepatic vein is dissected at this point.

The right and the middle hepatic veins are exposed. A cholecystectomy is then performed. After the right Glissonean pedicle is taped, the right hepatic artery and right portal vein are clipped and dissected. The right hepatic duct is dissected when parenchymal transection is performed and sufficient space is secured. Parenchymal transection is performed along the demarcation line. The Pringle maneuver [12] is employed if necessary. Deep parenchymal transection is performed with dorsal compression to control bleeding from the hepatic vein and inferior vena cava. The right hepatic vein is dissected with an endolineal stapling device, followed by dissection of the inferior vena cava ligament using an energy device. Right hepatectomy is then completed by dissecting the right triangular and coronary ligaments. The resected liver is bagged and retrieved from the Pfannenstiel incision.

#### 2.3.2. Left Hemihepatectomy

The patient is in the supine position. Trocar placement is shown in Figure 1. With dissection of the left triangular and coronary ligaments, cholecystectomy is performed. No mobilization of the left lateral section is performed. The left hepatic artery, middle hepatic artery, and left portal vein are dissected, and parenchymal dissection is performed along the demarcation line. After transection of the liver parenchyma, the left hepatic duct is dissected. Finally, the left hepatic vein is dissected using an endolineal stapling device, and the left hemihepatectomy is completed. The resected liver is bagged and retrieved from the Pfannenstiel incision.

## 3. Results

In this study, 15 patients who underwent TLhH were analyzed. Among these patients, TLhH was completed in 14 cases. One case had a conversion to a hand-assisted laparoscopic surgery because of the small body cavity volume and poor surgical view for pediatric patients (Table 1: Case 11).

Among the TLhH cases (11 men, 4 women), the median age was 63 years (range: 9–82 years). The preoperative diagnoses were HCC (*n* = 11), cystadenocarcinoma (*n* = 1), and benign disease (*n* = 3) (Table 1 and Table 2). There were no cases of intrahepatic cholangiocarcinoma (ICC) or metastatic liver tumor (Meta) in the giant tumor group (Table 2 and Table 3). The median tumor size (*n* = 15) was 12.0 cm (range: 10.5–15.0 cm) (Table 2). The types of hemihepatectomies consisted of: right (*n* = 12), extended left (*n* = 1), and left (*n* = 2) (Table 1). One patient who had HCC also showed liver cirrhosis (Table 2). All 15 patients had Child–Pugh grade A. The median operating time was 336 min (range: 175–441 min), and the median blood loss was 166 mL (range: 20–536 mL) (Table 2). One patient underwent a blood transfusion because of postoperative bleeding (Table 1: Case 1). The median postoperative hospital stay was 12 days (range: 7–117 days) (Table 2). There were no significant differences in the operating time, blood loss, and postoperative hospital stay between the nongiant tumor group (*n* = 65) and the giant tumor group (*n* = 15) (Table 2). There was no 90-day mortality, but one patient died of pneumonia as a result of prolonged bed rest associated with bile leakage (Table 1: Case 9).

Table 3 shows the surgical results of right hemihepatectomy. The median operating time, blood loss, and postoperative hospital stay in the giant tumor group were 327 min (range: 175–441 min), 151 mL (range: 20–450 mL), and 11 days (range: 7–117 days), respectively, and were not different to those in the nongiant tumor group (*n* = 23) (Table 3). The tumor size and weight of the resected liver were significantly larger in the giant tumor group (Table 3).

## 4. Discussion

There is consensus on the usefulness of laparoscopic liver resection for colorectal liver metastases and HCC [6]. Several metaanalyses and propensity score matching have reported that laparoscopic liver resection for colorectal liver metastasis improved short-term results compared to open liver resection, but showed no difference in long-term results [13,14]. A prospective randomized controlled trial also reported no difference in operating time and blood loss with laparoscopic liver resection and that there were significantly fewer postoperative complications [15]. For HCC, metaanalyses and propensity score matching have also reported reduced bleeding, shorter hospital stays, and fewer postoperative complications, but no difference in long-term results [16,17].

A propensity score matching report compared open and laparoscopic surgery in right hepatectomy for HCC, and showed reduced blood loss with laparoscopic surgery and no difference in long-term results [18]. In addition, it has been reported that the anterior approach has better short- and long-term results than the conventional approach in right hepatectomy for large HCCs [19,20]. There are several reports concerning laparoscopic hepatectomy for large liver cancer and these confirm that it can be performed safely, although the operating time is extended [8,9,10]. However, laparoscopic liver resection for large liver tumors is extremely difficult and is currently not commonly performed or performed only by experienced surgeons. The difficulty of laparoscopic liver resection for large tumors is due to the limited volume of the body cavity. A good surgical view cannot be ensured, and surgical operations are often difficult.

Conventional right hemihepatectomy performs hepatic parenchymal transection after liver mobilization. However, in the case of laparoscopic hepatectomy, liver mobilization cannot be performed first for giant liver tumors because of the limited volume of the body cavity. We have developed a safe laparoscopic right hemihepatectomy for giant liver tumors using an anterior approach. By creating a space on the right side of the inferior vena cava, retraction from the dorsal side is possible, enabling safe transection of the deep liver parenchyma, reducing the risk of major bleeding from the hepatic vein and inferior vena cava. It is a surgical procedure that can be performed safely by many surgeons, even in a narrow operative field. The procedure can also be performed in laparoscopic donor liver resections because a subcapsular hematoma can be avoided [21]. In addition, as a no-touch isolation technique, it can reduce the risk of tumors flowing into the inferior vena cava in cases such as hepatocellular carcinoma and colorectal liver metastasis. However, if the tumor invades the right side of the inferior vena cava or extends to the 12 o’clock point of the inferior vena cava, the indication is excluded. In addition, both left and right hepatectomy are excluded from indications if there is infiltration to multiple organs such as the diaphragm.

Liver hanging maneuvers in right hemihepatectomy have been reported in both open and laparoscopic procedures for safe anterior parenchymal transection [22,23]. In particular, Kim reported a modified liver hanging maneuver that involves placing tape between the inferior vena cava and the right adrenal gland [23]. There is no doubt about the usefulness of the hanging technique in right hemihepatectomy, but our method is a simple technique that compresses the liver parenchyma from the dorsal side. In the case of laparoscopic right hemihepatectomy for giant liver tumors, the narrow operating cavity may make the placement and hanging of the tape difficult, and our technique may be more suitable.

At our institution, laparoscopic hemihepatectomy in 15 cases with liver tumors larger than 10 cm was performed. The tumor diameters and resected liver volumes were significantly larger, but there were no differences in operating time, blood loss, complication rate, or postoperative hospital stay. In previous reports, the operating time was extended, but we were able to overcome this at our institution by devising the surgical technique [8,9,10]. Patients who underwent hemihepatectomy are not encouraged to leave the hospital early, so the postoperative hospital stay tends to be long. The median postoperative hospital stay was 12 days, but none of the postoperative stays were longer compared with the cases involving nongiant tumors. Experience of minor resection and major hepatectomy for nongiant tumors is necessary, but laparoscopic hemihepatectomy for giant tumors larger than 10 cm can be safely performed with limited indications. It is an operation that many surgeons can safely perform, and we hope that it will spread in the future.

## 5. Conclusions

Laparoscopic hemihepatectomy for giant tumors larger than 10 cm, in particular right tumors, can be performed safely. The surgical techniques have been standardized and are expected to spread widely in the future.

## Figures and Tables

**Table 1:** Individual clinical data of 15 patients who underwent totally laparoscopic hemihepatectomy with an anterior approach for giant liver tumors larger than 10 cm in diameter.

Case	Age/Sex	Disease	Types of Liver Resection	Conversion	Operating Time (min)	Blood Loss (mL)	Resected Tumor Size (cm)	Resected Liver Volume (g)	Complications (Clavien-Dindo ≥ III)	Postoperative Hospital Stay (Days)
1	78/M	HCC	Right		319	329	12.5	1250		38
2	59/M	HCC	Right		410	249	11.0	1200		8
3	48/M	HCC	Left		410	483	13.0	1250		12
4	60/F	Hemangioma	Right		390	155	11.5	1380		7
5	43/M	Hemangioma	Extended left		261	536	10.5	1050		14
6	74/M	HCC	Right		266	20	12.0	1100		14
7	75/M	HCC	Right		364	166	13.5	1592		7
8	61/F	Cyst	Right		175	101	15.0	1150		10
9	82/F	HCC	Right		357	122	13.6	1350	Bile leakage	117
10	63/M	HCC	Left		384	303	10.3	1000		13
11	9/M	HCC	Right	HALS	441	356	13.0	1100		9
12	69/M	HCC	Right		259	30	10.5	1180		9
13	64/M	HCC	Right		263	50	13.0	1340		12
14	73/M	HCC	Right		291	450	11.7	1260		15
15	57/F	Cystadeno-carcinoma	Right		336	148	12.0	1331		13

**Table 2:** Comparing the surgical outcomes for nongiant and giant liver tumors (≥10 cm) after laparoscopic hemihepatectomy.

	Nongiant Tumors (*n* = 65)	Giant Tumors (*n* = 15)	*p* Value ^†^
Age *	69 (26–85)	63 (9–82)	0.134
Disease	HCC 28, ICC 11, Meta 21, Benign 5	HCC 11, Cystadenocarcinoma 1, Benign 3	0.003 ^‡^
Liver cirrhosis	6	1	0.266 ^‡^
Types of hemihepatectomy Right/Left	23/42	12/3	0.004
Operating time (min) *	271 (165–559)	336 (175–441)	0.080
Blood loss (ml) *	72 (1–2473)	166 (20–536)	0.497
Resected tumor size (cm) *	4.0 (1.0–9.8)	12.0 (10.5–15.0)	<0.001
Resected liver volume (g) *	410 (150–800)	1250 (1000–1592)	<0.001
Postoperative hospital stay (days) *	12 (5–67)	12 (7–117)	0.496
Complications (Clavien-Dindo ≧ III)	9	1	0.664 ^‡^

* Values are median (range). † Student *t* test or ‡ χ^2^ test.

**Table 3:** Comparing the surgical outcomes for nongiant and giant liver tumors (≥10 cm) after laparoscopic right hemihepatectomy.

	Nongiant Tumors (*n* = 23)	Giant Tumors (*n* = 12)	*p* Value ^†^
Age *	69 (53–85)	67 (9–82)	0.320
Disease	HCC 13, ICC 4, Meta 6	HCC 9, Cystadenocarcinoma 1, Benign 2	0.022 ^‡^
Liver cirrhosis	2	0	0.443 ^‡^
Operating time (min) *	333 (181–559)	327 (175–441)	0.472
Blood loss (ml) *	153 (53–1232)	151 (20–450)	0.465
Resected tumor size (cm) *	4.5 (1.0–9.0)	12.3 (10.5–15.0)	<0.001
Resected liver volume (g) *	720 (390–800)	1255 (1100–1592)	<0.001
Postoperative hospital stay (days) *	13 (9–67)	11 (7–117)	0.976
Complications (Clavien-Dindo ≧ III)	6	1	0.373 ^‡^

* Values are median (range). † Student *t* test or ‡ χ^2^ test.

## Data Availability

The data presented in this study are available on request from the corresponding author.
